# Encouragement

**Published:** 1859-02

**Authors:** 


### ENCOURAGEMENT.

Some years ago a returned foreign missionary had almost settled down in the sad conclusion that for the remainder of a life yet young, he was to be but a cumberer of the ground; but a letter just received says—" I am happy to say that my health is now unusually good; I am under the necessity of being constantly vigilant; yet, with due caution, I labor hard, as hard as any of my brethren, and, what is far better, it awakens my sincerest gratitude God has greatly blessed my labors. For all this, under Him, I am indebted, my dear sir, to you; and that He may make you the instrument of still more and more good, especially in helping his poor broken ministers, is my sincere desire," &c.

There is a lesson of the very highest importance in this narration. This gentleman was enabled to maintain his ability for pastoral labor, hard but successful, by means of constant, untiring vigilance. Very many attempt to test the perfection of their cure by unnecessary exposures or extravagances; others by the most unpardonable indifference or inattention to their health, with the result of coming back to the physician with almost expressed upbraidings for a "temporary" improvement. The price of life to any one who has been seriously ill is eternal vigilance.

